# The SMuRF-less paradox in ST-segment-elevation myocardial infarction: a retrospective hospital-based two-cohort study

**DOI:** 10.3389/fcvm.2026.1955087

**Published:** 2026-10-30

**Authors:** Jing Zhao, Junchu Wei, Chenliang Pan, Yu Peng, Peng Lei, Qiguo Lian, Jun Lyu, Ming Bai, Zheng Zhang

**Affiliations:** 1 The First School of Clinical Medicine, Lanzhou University, Lanzhou, China; 2 Department of Cardiology, The First Hospital of Lanzhou University, Lanzhou, China; 3 Gansu Province Clinical Research Centre for Cardiovascular Diseases, The First Hospital of Lanzhou University, Lanzhou, China; 4 Shanghai-MOST Key Laboratory of Health and Disease Genomics, Shanghai Institute for Biomedical and Pharmaceutical Technologies, Shanghai, China; 5 Clinical Research Center, The First Affiliated Hospital of Jinan University, Guangzhou, China

**Keywords:** cohort study, mortality, prognosis, SMuRF-less, STEMI

## Abstract

**Background:**

Patients with ST-segment-elevation myocardial infarction (STEMI) without standard modifiable cardiovascular risk factors (SMuRFs) exhibit paradoxically increased mortality. We aimed to characterize associations with adverse outcomes and identify potential drivers.

**Methods:**

We included patients with STEMI from a hospital-based cohort in Lanzhou, China (*n* = 6,892; 2014–2023) and the US Medical Information Mart for Intensive Care IV (MIMIC-IV; *n* = 4,480; 2008–2019). SMuRF-less status was defined as the absence of hypertension, diabetes, hyperlipidemia, and smoking, with obesity included in sensitivity analyses. Primary outcomes were 30- and 365-day all-cause mortality. Secondary outcomes were major adverse cardiac and cerebrovascular events (MACCEs), 7-day delirium and acute kidney injury (AKI). Associations were estimated using adjusted hazard ratios (aHRs) with 95% confidence intervals (CIs), and Fine–Gray competing-risk analyses performed for 7-day delirium and AKI.

**Results:**

SMuRF-less patients comprised 17.2% (*n* = 1,182) of the Lanzhou cohort and 13.6% (*n* = 608) of the MIMIC-IV cohort. SMuRF-less status was associated with higher 30-day mortality in both cohorts (Lanzhou: aHR, 1.37; 95% CI, 1.09–1.71; MIMIC-IV: 1.67, 1.39–2.01). In landmark analyses, SMuRF-less status was not significantly associated with mortality during days 31–365 in Lanzhou (1.68; 0.84–3.37) but was associated with higher mortality in MIMIC-IV (1.34; 1.02–1.77). SMuRF-less status was not significantly associated with MACCEs in Lanzhou. In MIMIC-IV, SMuRF-less status was associated with a higher risk of 7-day delirium (1.40; 1.17–1.67) but was not associated with 7-day AKI (1.05; 0.94–1.17). For both 7-day delirium and AKI, competing-risk analyses yielded consistent findings. In subgroup analyses, excess 30-day mortality was greater among patients in Lanzhou not receiving extracorporeal membrane oxygenation, those not receiving intra-aortic balloon pump, and those with Killip class I–III (*P* for interaction <0.05). Sensitivity analyses incorporating obesity showed similar associations for 30-day mortality in Lanzhou (1.40; 1.12–1.76) and MIMIC-IV (1.63; 1.33–1.99), as well as for 7-day delirium (1.33; 1.09–1.62).

**Conclusions:**

SMuRF-less status was independently associated with increased early mortality in both cohorts and a higher risk of delirium in the MIMIC-IV cohort. This excess risk was not fully explained by measured clinical and physiological markers, suggesting that residual risk may extend beyond conventional modifiable cardiovascular risk factors.

## 1 Introduction

ST-segment-elevation myocardial infarction (STEMI) remains a major cause of cardiovascular mortality and continues to impose a substantial burden worldwide, including in China and the United States (1, 2). Traditionally, clinical risk stratification and secondary prevention strategies have been anchored in the identification of four standard modifiable cardiovascular risk factors (SMuRFs): hypertension, diabetes mellitus, hyperlipidemia, and smoking (3). However, approximately 10%–25% of patients with STEMI have none of these conventional risk factors and are termed “SMuRF-less” (4, 5). Paradoxically, emerging studies suggest that SMuRF-less patients may experience excess mortality, although findings remain inconsistent (5, 6). This observation challenges the traditional Framingham-based paradigm, which posits that a lower burden of risk factors is associated with a more favorable prognosis (7).

Previous studies of the SMuRF-less paradox have mainly focused on mortality, with limited assessment of other adverse outcomes (8, 9). Moreover, obesity, an important metabolic risk factor, has not been adequately considered when defining this phenotype, particularly in Chinese populations (10). The mechanisms underlying the excess risk in SMuRF-less patients also remain unclear. Further investigation is therefore needed to determine whether clinical presentations, hemodynamic instability, and treatment intensity may partly explain the observed associations (11).

In this study, we conducted a large-scale, retrospective hospital-based dual-cohort analysis. We used data from the Lanzhou STEMI cohort in China and the Medical Information Mart for Intensive Care IV (MIMIC-IV) database in the United States. Our objectives were to (1) compare adverse outcomes, including mortality and acute complications, between SMuRF-less and SMuRF patients; (2) characterize early and late mortality risks using a 30-day landmark analysis; and (3) perform sequential attenuation analyses to explore potential pathways underlying the SMuRF-less paradox.

## 2 Methods

### 2.1 Study design, data sources, and population

The Lanzhou STEMI cohort was established based on the China STEMI Care Project (CSCAP) and integrated with data from the National Chest Pain Centers Program (NCPCP) and electronic health records (EHRs) (12). The Lanzhou cohort enrolled consecutive patients with STEMI admitted to the Heart Center of the First Hospital of Lanzhou University (LZUFH) between January 1, 2014, and December 31, 2023. Eligibility was defined according to the Fourth Universal Definition of Myocardial Infarction and the Chinese diagnostic criteria for STEMI (13, 14). Follow-up records were obtained from the LZUFH, and follow-up was defined from hospital admission until death or the last documented medical encounter or telephone contact. A total of 6,892 patients were included in the final analysis (Figure 1A). The design of MIMIC-IV has been reported previously (15, 16). In the MIMIC-IV cohort, patients with STEMI were identified using International Classification of Diseases, Ninth Revision (ICD-9) codes 410.x (excluding 410.7x) and Tenth Revision (ICD-10) diagnosis codes I21.x (excluding I21.4). A total of 4,480 patients were included in the MIMIC-IV cohort (Figure 1B). The ethics committee of the First Hospital of Lanzhou University approved the study and waived the requirement for informed consent (Nos. LDYYLL2015-0080 and LDYYLL2022-304).

**Figure 1:**
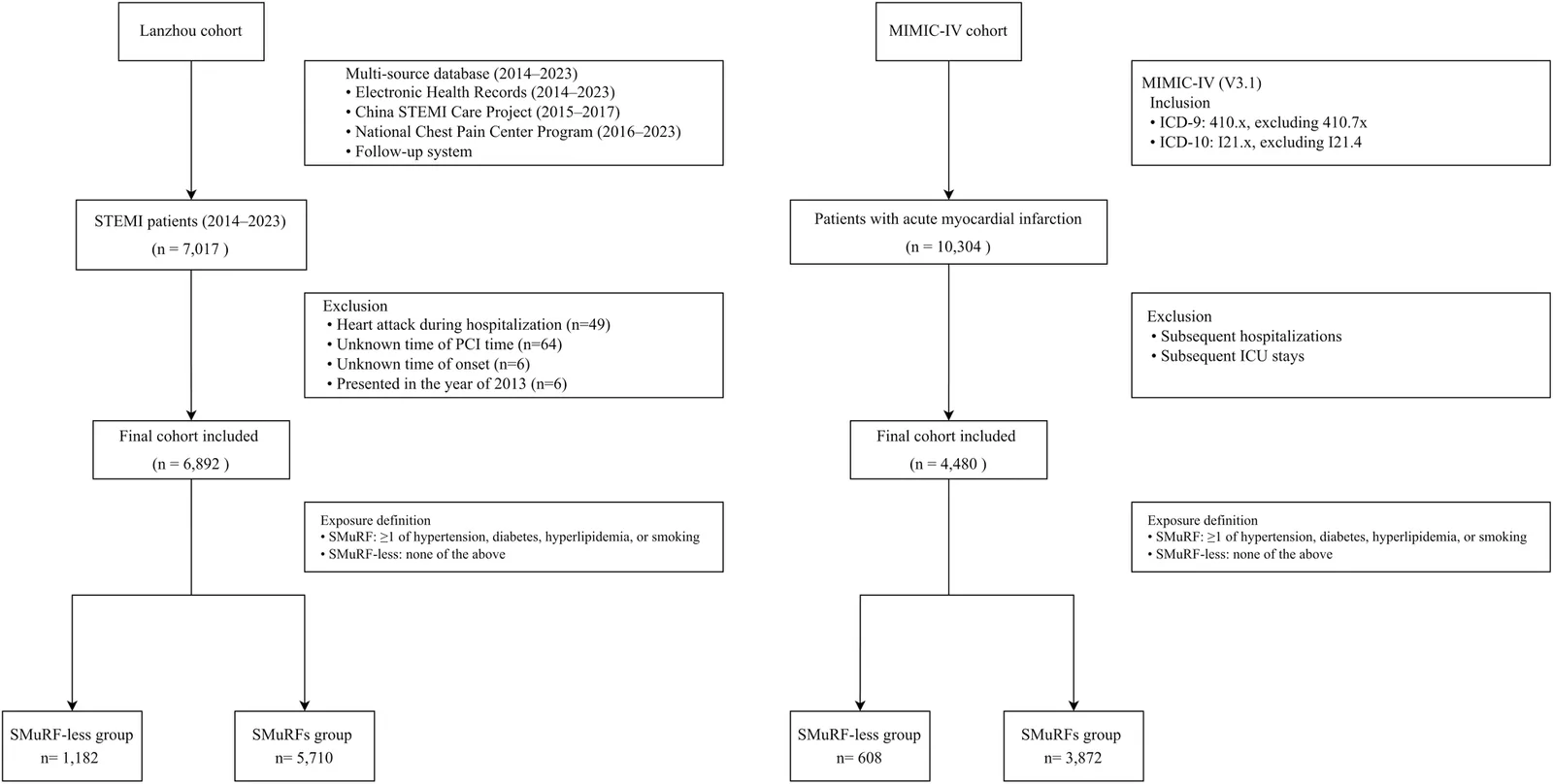
Flowchart of patients included in two cohorts. STEMI, ST-segment–elevation myocardial infarction; SMuRF, standard modifiable cardiovascular risk factors.

### 2.2 Exposure assessment

SMuRF status was determined using EHRs. Hypertension was ascertained from documented medical history or diagnosis during the index admission. Diabetes mellitus was defined by medical history or diagnosis during the index admission. Hyperlipidemia was defined by medical history, known low-density lipoprotein cholesterol >135 mg/dL (3.5 mmol/L) or total cholesterol >213 mg/dL (5.5 mmol/L), or diagnosis during the index admission. Smoking status was extracted from medical history records and defined as smoking at least one cigarette per day for more than six consecutive months. SMuRF-less status was defined as the absence of all four factors. Body mass index (BMI) was calculated as weight in kilograms divided by the square of height in meters (kg/m^2^). In the sensitivity analysis, obesity (BMI ≥30 kg/m^2^) was included as an additional risk factor, and thus SMuRF-less status was defined as the absence of all five factors.

### 2.3 Covariates

In the Lanzhou cohort, variables included sociodemographic characteristics, medical history, clinical measurements at admission, medical treatments, and interventions. Primary percutaneous coronary intervention (PCI) was defined as PCI performed within 48 h of symptom onset. Early reperfusion was defined as receiving reperfusion therapy (either primary PCI or thrombolysis) within 48 h. Total ischemic time was defined as the interval from symptom onset to reperfusion. In the MIMIC-IV cohort, 117 candidate variables were extracted based on clinical evidence and data availability. These included demographics, vital signs, comorbidities, medical treatments, organ support measures, and laboratory parameters, including complete blood count, blood gas analysis, cardiac biomarkers, renal function, electrolytes, and coagulation profiles.

### 2.4 Outcome ascertainment

In the Lanzhou cohort, the primary outcomes were 30-day and 365-day all-cause mortality. Secondary outcomes included in-hospital major adverse cardiac and cerebrovascular events (MACCEs), defined as new-onset heart failure, non-fatal stroke, non-fatal reinfarction, and non-fatal target vessel revascularization (TVR). In the MIMIC-IV cohort, the primary outcomes were also 30-day and 365-day all-cause mortality. The secondary outcomes were 7-day delirium and 7-day acute kidney injury (AKI) (17). In the sensitivity analysis, we additionally evaluated major adverse kidney events within 30 days (MAKE30), defined as a composite of all-cause death, initiation of new renal replacement therapy, or persistent renal dysfunction (final serum creatinine ≥200% of the baseline value) within 30 days after admission (18).

### 2.5 Statistical analysis

All statistical analyses were performed using Stata SE 15.1 (StataCorp LLC, College Station, Texas, USA) and R 4.4.0 (R Foundation for Statistical Computing). All tests were two-sided, with *p* < 0.05 considered statistically significant. In the Lanzhou cohort, missingness for covariates was low, including BMI (7.54%), systolic blood pressure (0.25%), heart rate (2.67%), creatinine (3.48%), and length of stay (0.15%). In the MIMIC-IV cohort, variables with >35% missingness were excluded from analysis. Multiple imputation by chained equations was performed, and the first imputed dataset was used for the primary analyses. In the MIMIC-IV cohort, multistep variable selection was used to optimize the analysis. First, after excluding 32 variables with high missingness, eight variables were retained as mandatory variables based on *a priori* clinical knowledge. Second, five redundant variables were removed to reduce multicollinearity. Third, variable selection was performed among the remaining candidates using least absolute shrinkage and selection operator (LASSO) regression with 200 bootstrap resamples, retaining the most frequently selected variable within each functional category (19). Fourth, after excluding one non-biological metric (glucose monitoring frequency), 53 variables were included for subsequent analysis.

Kaplan–Meier curves and a 30-day landmark analysis were used to distinguish early and late mortality risks. Associations between SMuRF status and outcomes were evaluated using logistic regression, linear regression, and Cox proportional hazards models. Effect estimates were reported as odds ratios (ORs), beta coefficients, or hazard ratios (HRs) with 95% confidence intervals (CIs), as appropriate. Three models were constructed as follows: in the Lanzhou cohort, model 1 was unadjusted; model 2 was adjusted for demographics (age, sex, ethnicity, insurance, and residence), comorbidities [cardiovascular past medical history, chronic obstructive pulmonary disease (COPD)], and clinical presentation [systolic blood pressure (SBP), heart rate, creatinine, infarct location, cardiogenic shock, and Killip class IV]; and model 3 was further adjusted for acute treatments, including aspirin use, P2Y12 inhibitor use, timely reperfusion, intra-aortic balloon pump (IABP), and extracorporeal membrane oxygenation (ECMO). In the MIMIC-IV cohort, model 1 was unadjusted; model 2 was adjusted for demographics (age, sex, race, and insurance) and severity-related variables [Charlson comorbidity index (CCI), SBP, heart rate, respiratory rate, and creatinine]; and model 3 was further adjusted for treatment variables, including aspirin use, P2Y12 inhibitor use, PCI, mechanical ventilation, IABP, and ECMO. Subgroup and interaction analyses were performed using Cox models, and the results were presented as forest plots. In the MIMIC-IV cohort, sequential attenuation analyses were performed to explore potential pathways underlying the associations. Covariates were added sequentially to the Cox model, and the percentage change in the β coefficient was calculated at each step. Additionally, competing-risk analyses were performed using Fine–Gray subdistribution hazards models, with death treated as a competing event for AKI and delirium. Multinomial logistic regression was used to evaluate the individual components of MAKE30 in the MIMIC-IV cohort. Exploratory analyses were conducted to assess sex-related differences in age and mortality among SMuRF-less patients.

## 3 Results

### 3.1 Population characteristics and clinical presentation

A total of 6,892 patients with STEMI from the Lanzhou cohort and 4,480 patients with STEMI from the MIMIC-IV database were included in the final analysis. SMuRF-less status was identified in 1,182 (17.2%) and 608 (13.6%) individuals in the Lanzhou and MIMIC-IV cohorts, respectively. For SMuRFs, the distribution of risk factors differed between the two cohorts. In the Lanzhou cohort, current smoking was the most prevalent risk factor (66.9%), followed by hypertension (51.0%), diabetes mellitus (22.2%), and hyperlipidemia (20.1%). The MIMIC-IV cohort showed a distinct risk profile dominated by metabolic conditions, with hypertension being the most common (74.2%), followed by hyperlipidemia (66.8%) and diabetes mellitus (46.3%), while smoking prevalence was notably lower (13.2%).

Demographic and clinical characteristics of both the Lanzhou and MIMIC-IV cohorts are presented in Table 1. In the Lanzhou cohort, compared with patients with SMuRFs, those in the SMuRF-less group were older and more likely to be female or of ethnic minority origin (all *P* < 0.05). There were no significant differences in COPD or cardiovascular past medical history between groups. In the MIMIC-IV cohort, SMuRF-less patients were younger and more likely to have private medical insurance (all *P* < 0.05). SMuRF-less patients also had a lower burden of comorbidities, including lower CCI scores and a lower prevalence of COPD, cardiovascular past medical history, atrial fibrillation, congestive heart failure, valvular heart disease, peripheral vascular disease, and chronic kidney disease (all *P* < 0.05).

**Table 1:** Baseline characteristics.

Variables	Total	SMuRF-less	SMuRF	*P*-value
	*n* = 6,892	*n* = 1,182	*n* = 5,710	
Lanzhou cohort				
SMuRFs *n* (%)				
Smoking history	3,819 (55.4%)	0 (0.0%)	3,819 (66.9%)	<0.001
Diabetes mellitus	1,269 (18.4%)	0 (0.0%)	1,269 (22.2%)	<0.001
Hypertension	2,912 (42.3%)	0 (0.0%)	2,912 (51.0%)	<0.001
Hyperlipidemia	1,148 (16.7%)	0 (0.0%)	1,148 (20.1%)	<0.001
Demographics				
Age, years	61 (11)	62 (12)	60 (11)	<0.001
Male	5,874 (85.2%)	898 (76.0%)	4,976 (87.1%)	<0.001
Han ethnicity	6,349 (92.1%)	1,069 (90.4%)	5,280 (92.5%)	0.018
Employed-insurance	2,580 (37.4%)	408 (34.5%)	2,172 (38.0%)	0.023
Urban residency	2,596 (37.7%)	446 (37.7%)	2,150 (37.7%)	0.960
Other risk factors				
BMI (kg/m^2^)	24.22 (21.97–26.30)	23.67 (21.48–25.91)	24.22 (21.97–26.47)	<0.001
BMI category				
Underweight	253 (3.7%)	55 (4.7%)	198 (3.5%)	<0.001
Normal	3,008 (43.6%)	574 (48.6%)	2,434 (42.6%)	
Overweight	2,781 (40.4%)	444 (37.6%)	2,337 (40.9%)	
Obese	850 (12.3%)	109 (9.2%)	741 (13.0%)	
Cardiovascular past medical history	583 (8.5%)	88 (7.4%)	495 (8.7%)	0.170
COPD	70 (1.0%)	11 (0.9%)	59 (1.0%)	0.750
Clinical characteristics				
SBP, mmHg	126 (24)	120 (24)	127 (24)	<0.001
HR, beats/min	79 (17)	80 (19)	79 (17)	0.420
Creatinine (μmol/L)	73 (63–86)	72 (61–84)	73 (64–86)	0.007
Killip class IV	545 (7.9%)	163 (13.8%)	382 (6.7%)	<0.001
Cardiogenic shock	446 (6.5%)	144 (12.2%)	302 (5.3%)	<0.001
Locations of MI				
Anterior	3,527 (51.2%)	636 (53.8%)	2,891 (50.6%)	0.047
Inferior	2,887 (41.9%)	467 (39.5%)	2,420 (42.4%)	0.068
Lateral	236 (3.4%)	44 (3.7%)	192 (3.4%)	0.540
Posterior	160 (2.3%)	28 (2.4%)	132 (2.3%)	0.910
Right ventricular	53 (0.8%)	9 (0.8%)	44 (0.8%)	0.970
Guideline-based medication treatment				
Initial aspirin	6,744 (97.9%)	1,148 (97.1%)	5,596 (98.0%)	0.057
Initial P2Y12i	6,834 (99.2%)	1,164 (98.5%)	5,670 (99.3%)	0.005
Statin in hospital	6,780 (98.4%)	1,133 (95.9%)	5,647 (98.9%)	<0.001
β-block in hospital	4,866 (70.6%)	789 (66.8%)	4,077 (71.4%)	0.001
Other life support				
IABP	547 (7.9%)	125 (10.6%)	422 (7.4%)	<0.001
ECMO	152 (2.2%)	45 (3.8%)	107 (1.9%)	<0.001
Temporary pacing	403 (5.8%)	72 (6.1%)	331 (5.8%)	0.690
CRRT	90 (1.3%)	15 (1.3%)	75 (1.3%)	0.900
Coronary intervention strategy				
Initial reperfusion strategy				
None	2,102 (30.5%)	359 (30.4%)	1,743 (30.5%)	0.980
Thrombolytic therapy	1,556 (22.6%)	265 (22.4%)	1,291 (22.6%)	
Primary PCI	3,234 (46.9%)	558 (47.2%)	2,676 (46.9%)	
Final reperfusion strategy				
Primary PCI	4,610 (66.9%)	798 (67.5%)	3,812 (66.8%)	0.620
Thrombolysis + primary PCI	1,376 (20.0%)	240 (20.3%)	1,136 (19.9%)	0.750
None of any interventional procedure	388 (5.6%)	92 (7.8%)	296 (5.2%)	<0.001
None of any reperfusion therapy	359 (5.2%)	83 (7.0%)	276 (4.8%)	0.002
Stent implantation	5,708 (82.8%)	900 (76.1%)	4,808 (84.2%)	<0.001
Delays in admission				
Symptoms-to-fmc				
<2 h	1,577 (22.9%)	282 (23.9%)	1,295 (22.7%)	0.380
Symptoms-to-thrombolysis				
<3 h	568 (8.2%)	92 (7.8%)	476 (8.3%)	0.530
Symptoms-to-device				
<2 h	145 (2.1%)	22 (1.9%)	123 (2.2%)	0.520
Symptoms -to-start of reperfusion therapy				
<3 h	963 (14.0%)	152 (12.9%)	811 (14.2%)	0.230
<12 h	3,167 (46.0%)	541 (45.8%)	2,626 (46.0%)	0.890
Symptoms-to-door of PCI hospital				
<24 h	4,659 (67.6%)	828 (70.1%)	3,831 (67.1%)	0.048
Variables	Total	SMuRF-less	SMuRF	*P*-value
	*N* = 4,480	*N* = 608	*N* = 3,872	
MIMIC-IV cohort.				
SMuRFs, *n* (%)				
Smoking history	512 (11.4%)	0 (0.0%)	512 (13.2%)	<0.001
Diabetes mellitus	1,791 (40.0%)	0 (0.0%)	1,791 (46.3%)	<0.001
Hypertension	2,872 (64.1%)	0 (0.0%)	2,872 (74.2%)	<0.001
Hyperlipidemia	2,585 (57.7%)	0 (0.0%)	2,585 (66.8%)	<0.001
BMI (kg/m^2^)	27.6 (24.1–31.6)	26.5 (23.5–30.6)	27.8 (24.2–31.8)	<0.001
BMI category				
Underweight	97 (2.2%)	16 (2.6%)	81 (2.1%)	0.001
Normal	1,315 (29.4%)	212 (34.9%)	1,103 (28.5%)	
Overweight	1,544 (34.5%)	211 (34.7%)	1,333 (34.4%)	
Obese	1,524 (34.0%)	169 (27.8%)	1,355 (35.0%)	
Demographics				
Age, years	71 (13)	66 (16)	72 (13)	<0.001
Male	2,846 (63.5%)	393 (64.6%)	2,453 (63.4%)	0.540
Race/ethnicity				
White	2,838 (63.3%)	369 (60.7%)	2,469 (63.8%)	<0.001
Black	344 (7.7%)	25 (4.1%)	319 (8.2%)	
Hispanic	105 (2.3%)	10 (1.6%)	95 (2.5%)	
Asian	105 (2.3%)	14 (2.3%)	91 (2.4%)	
Other or unknown	1,088 (24.3%)	190 (31.2%)	898 (23.2%)	
Insurance type				
Private insurance	1,030 (23.0%)	185 (30.4%)	845 (21.8%)	<0.001
Medicare	2,861 (63.9%)	304 (50.0%)	2,557 (66.0%)	
Medicaid	414 (9.2%)	78 (12.8%)	336 (8.7%)	
Other or unknown	175 (3.9%)	41 (6.7%)	134 (3.5%)	
Marital status				
Married	2,458 (54.9%)	336 (55.3%)	2,122 (54.8%)	0.830
Not married	2,022 (45.1%)	272 (44.7%)	1,750 (45.2%)	
Comorbidities				
Charlson comorbidity index	6.0 (4.0–8.0)	4.0 (3.0–6.0)	6.0 (5.0–8.0)	<0.001
COPD	1,087 (24.3%)	123 (20.2%)	964 (24.9%)	0.013
Cardiovascular past medical history	2,107 (47.0%)	131 (21.5%)	1,976 (51.0%)	<0.001
Atrial fibrillation	1,116 (24.9%)	97 (16.0%)	1,019 (26.3%)	<0.001
Congestive heart failure	2,249 (50.2%)	195 (32.1%)	2,054 (53.0%)	<0.001
Valvular heart disease	899 (20.1%)	73 (12.0%)	826 (21.3%)	<0.001
Peripheral vascular disease	679 (15.2%)	63 (10.4%)	616 (15.9%)	<0.001
Chronic kidney disease	1,281 (28.6%)	40 (6.6%)	1,241 (32.1%)	<0.001
Anemia	1,041 (23.2%)	134 (22.0%)	907 (23.4%)	0.450
Peptic ulcer disease	130 (2.9%)	14 (2.3%)	116 (3.0%)	0.340
Thyroid disease	586 (13.1%)	68 (11.2%)	518 (13.4%)	0.140
Malignancy	364 (8.1%)	59 (9.7%)	305 (7.9%)	0.130
Clinical characteristics and labs				
Vital signs				
SBP, mmHg	88.5 (17.3)	86.4 (17.7)	88.9 (17.2)	<0.001
Heart rate, bpm	101 (21)	105 (23)	101 (20)	<0.001
Respiratory rate, breaths/min	19.7 (3.7)	20.2 (4.1)	19.6 (3.6)	<0.001
MAP, mmHg	58.4 (14.0)	58.8 (15.5)	58.3 (13.8)	0.440
Lactate, mmol/L	2.30 (1.60–3.60)	2.40 (1.60–3.95)	2.30 (1.60–3.60)	0.095
Cardiac markers				
CK-MB, U/L	23.0 (8.0–92.0)	36.0 (11.0–151.5)	22.0 (8.0–85.0)	<0.001
Renal function				
Creatinine, mg/dL	1.20 (0.90–2.00)	1.10 (0.90–1.60)	1.20 (0.90–2.00)	<0.001
BUN, mg/dL	20.0 (14.0–34.0)	17.0 (13.0–27.0)	21.0 (14.0–36.0)	<0.001
Electrolytes				
Calcium, mg/dL	8.7 (8.2–9.1)	8.6 (8.1–9.0)	8.7 (8.2–9.1)	<0.001
Sodium, mmol/L	140.0 (137.0–142.0)	140.0 (138.0–143.0)	140.0 (137.0–142.0)	<0.001
Potassium, mmol/L	3.9 (3.6–4.3)	3.9 (3.5–4.3)	3.9 (3.6–4.3)	0.017
Chloride, mmol/L	106.0 (102.0–109.0)	106.0 (103.0–109.0)	105.0 (102.0–109.0)	0.002
Bicarbonate, mmol/L	21.0 (18.0–24.0)	21.0 (17.0–23.0)	21.0 (18.0–24.0)	<0.001
Coagulation				
INR	1.2 (1.1–1.3)	1.2 (1.1–1.4)	1.2 (1.1–1.3)	0.002
PT	13.0 (11.9–14.7)	13.10 (12.0–15.1)	13.1 (11.9–14.7)	0.043
Blood count				
Platelet count, ×10^9^/L	221 (173–281)	224 (171–286)	221 (173–280)	0.660
WBC, ×10^9^/L	9.9 (7.5–13.1)	10.5 (7.8–14.0)	9.9 (7.5–12.8)	0.003
Hemoglobin, g/dL	11.8 (10.2–13.5)	12.3 (10.6–13.8)	11.8 (10.2–13.4)	<0.001
Hematocrit, %	31.5 (26.3–36.7)	32.7 (27.4–37.8)	31.3 (26.1–36.5)	<0.001
Metabolic markers				
Glucose, mg/dL	96.0 (85.0–113.0)	94.0 (85.0–108.0)	97.0 (85.0–114.0)	0.044
Anion gap, mmol/L	13.0 (11.0–15.0)	12.0 (10.0–14.5)	13.0 (11.0–15.0)	<0.001
Medical treatment				
Aspirin	3,922 (87.5%)	466 (76.6%)	3,456 (89.3%)	<0.001
P2Y12i	2,283 (51.0%)	277 (45.6%)	2,006 (51.8%)	0.004
Statin	3,825 (85.4%)	426 (70.1%)	3,399 (87.8%)	<0.001
ACEi	1,983 (44.3%)	240 (39.5%)	1,743 (45.0%)	0.011
ARB	515 (11.5%)	25 (4.1%)	490 (12.7%)	<0.001
Beta-blocker	3,783 (84.4%)	441 (72.5%)	3,342 (86.3%)	<0.001
SGLT2i	18 (0.4%)	1 (0.2%)	17 (0.4%)	0.320
PPI	2,230 (49.8%)	274 (45.1%)	1,956 (50.5%)	0.012
Early vasopressor use	193 (4.3%)	40 (6.6%)	153 (4.0%)	0.003
Procedures				
PCI	1,412 (31.5%)	182 (29.9%)	1,230 (31.8%)	0.370
CABG	819 (18.3%)	60 (9.9%)	759 (19.6%)	<0.001
Early mechanical ventilation	1,668 (37.2%)	252 (41.4%)	1,416 (36.6%)	0.021
ECMO	40 (0.9%)	12 (2.0%)	28 (0.7%)	0.002
IABP	468 (10.4%)	59 (9.7%)	409 (10.6%)	0.520
CRRT	405 (9.0%)	43 (7.1%)	362 (9.3%)	0.069

ACEi, angiotensin-converting enzyme inhibitor; ARB, angiotensin receptor blocker; BMI, body mass index; BUN, blood urea nitrogen; CABG, coronary artery bypass grafting; CK-MB, creatine kinase-MB; COPD, chronic obstructive pulmonary disease; CRRT, continuous renal replacement therapy; ECMO, extracorporeal membrane oxygenation; IABP, intra-aortic balloon pump; INR, international normalized ratio; MAP, mean arterial pressure; MI, myocardial infarction; PCI, percutaneous coronary intervention; PPI, proton pump inhibitor; PT, prothrombin time; P2Y12i, P2Y12 inhibitor; SBP, systolic blood pressure; SGLT2i, sodium–glucose cotransporter-2 inhibitor; SMuRF, standard modifiable cardiovascular risk factor; WBC, white blood cell count.

Despite a lower burden of modifiable risk factors, SMuRF-less patients in both cohorts presented with more severe clinical features. In the Lanzhou cohort, the SMuRF-less group was characterized by lower SBP and higher proportions of anterior myocardial infarction, Killip class IV, and cardiogenic shock (all *P* < 0.05). Similarly, in the MIMIC-IV cohort, SMuRF-less patients had lower SBP, higher heart and respiratory rates, and higher creatine kinase-MB (CK-MB) levels (36.0 vs. 22.0 U/L). The SMuRF-less group also had higher white blood cell counts (10.5 vs. 9.9 × 10^9^/L), hemoglobin levels (12.3 vs. 11.8 g/dL), hematocrit levels (32.7% vs. 31.3%), and chloride levels (106.0 vs. 105.0 mmol/L) (all *P* < 0.01). Notably, SMuRF-less patients in both cohorts had lower creatinine levels at admission (all *P* < 0.05).

### 3.2 Procedural interventions and medical management

In the Lanzhou cohort, SMuRF-less patients were less likely to receive timely reperfusion therapy and had higher rates of no reperfusion therapy (7.0% vs. 4.8%; *P* < 0.001) and no interventional procedures (7.8% vs. 5.2%; *P* < 0.001). Stent implantation was also less frequent in the SMuRF-less group (76.1% vs. 84.2%; *P* < 0.001). Similarly, in the MIMIC-IV cohort, SMuRF-less status was associated with a lower rate of coronary artery bypass grafting (CABG; 9.9% vs. 19.6%; *P* < 0.05), whereas PCI rates were comparable between groups (29.9% vs. 31.8%).

Regarding medical therapy, SMuRF-less patients in the Lanzhou cohort were less likely to receive guideline-directed medical therapy (GDMT), including P2Y12 inhibitors (98.5% vs. 99.3%), statins (95.8% vs. 98.9%), and β-blockers (66.8% vs. 71.4%) (all *P* < 0.01), while aspirin use was numerically lower (97.1% vs. 98.0%; *P* = 0.057). This pattern was more pronounced in the MIMIC-IV cohort, where SMuRF-less patients had lower rates of in-hospital aspirin (76.6% vs. 89.3%), P2Y12 inhibitors (45.6% vs. 51.8%), statins (70.1% vs. 87.8%), angiotensin-converting enzyme inhibitors (39.5% vs. 45.0%), angiotensin receptor blockers (4.1% vs. 12.7%), proton pump inhibitors (45.1% vs. 50.5%), and β-blockers (72.5% vs. 86.3%) (all *P* < 0.05). In contrast, SMuRF-less patients were more likely to receive advanced life support. In the Lanzhou cohort, use of IABP (10.6% vs. 7.4%) and ECMO (3.8% vs. 1.9%) was higher among SMuRF-less patients (both *P* < 0.001). Likewise, in the MIMIC-IV cohort, SMuRF-less patients had higher rates of early vasopressor use (6.6% vs. 4.0%), mechanical ventilation (41.4% vs. 36.6%), and ECMO use (2.0% vs. 0.7%; all *P* < 0.05).

Most treatment-delay metrics in the Lanzhou cohort were comparable between groups (Supplementary Table S1). No significant differences were observed in the proportions of patients achieving symptom-to-first medical contact within 2 h, symptom-to-thrombolysis within 3 h, or symptom-to-wire within 2 h. SMuRF-less patients were slightly more likely to reach a PCI-capable center within 24 h (70.1% vs. 67.1%). Among patients undergoing primary PCI, analysis of nine delay-related metrics showed that symptom-to-device time was longer in the SMuRF-less group (456 vs. 425 min; *P* = 0.006), whereas the other eight metrics showed no significant differences.

### 3.3 Association between SMuRF-less status and mortality

SMuRF-less status was associated with higher mortality in both cohorts (Table 2). In the Lanzhou cohort, 30-day mortality was higher among SMuRF-less patients than among those with SMuRFs (10.1% vs. 4.1%; *P* < 0.001), with a similar pattern in the MIMIC-IV cohort (26.2% vs. 18.8%; *P* < 0.05). Kaplan–Meier curves showed early separation between the groups. Landmark analyses further indicated that the association between SMuRF-less status and mortality was stronger during the first 30 days after admission (Figures 2A–D). After multivariable adjustment, SMuRF-less status was associated with higher 30-day mortality in both the Lanzhou cohort (adjusted hazard ratio [aHR], 1.37; 95% CI, 1.09–1.71) and the MIMIC-IV cohort (aHR, 1.67; 95% CI, 1.39–2.01). Among patients in the Lanzhou cohort who survived to day 30, 11 of 899 SMuRF-less patients (1.2%) and 34 of 4,683 patients with SMuRFs (0.7%) died between days 31 and 365. SMuRF-less status was not significantly associated with mortality during this period (aHR, 1.68; 95% CI, 0.84–3.37; *P* = 0.144). However, in the MIMIC-IV cohort, mortality between days 31 and 365 was 13.6% among SMuRF-less patients and 16.8% among patients with SMuRFs. After multivariable adjustment, SMuRF-less status was significantly associated with higher mortality (aHR, 1.34; 95% CI, 1.02–1.77; *P* = 0.037).

**Table 2:** Association of SMuRF-less status With mortality and clinical outcomes in the lanzhou and MIMIC-IV cohorts.

Outcomes	SMuRF-less (*n*, %)	SMuRF (*n*, %)	Hazard ratio (95% CI)^a^					
			Model 1	*P*-value	Model 2	*P*-value	Model 3	*P*-value
30-day mortality								
Lanzhou cohort	119/1,182 (10.1%)	233/5,710 (4.1%)	2.58 (2.07–3.22)	<0.001	1.42 (1.14–1.78)	0.002	1.37 (1.09–1.71)	0.007
MIMIC-IV cohort	159/608 (26.2%)	726/3,872 (18.8%)	1.48 (1.25–1.76)	<0.001	1.85 (1.55–2.22)	<0.001	1.67 (1.39–2.01)	<0.001
31–365 day mortality								
Lanzhou cohort	11/899 (1.2%)	34/4,683 (0.7%)	1.75 (0.88–3.45)	0.108	1.62 (0.81–3.24)	0.175	1.68 (0.84–3.37)	0.144
MIMIC-IV cohort	61/449 (13.6%)	530/3,146 (16.8%)	0.80 (0.61–1.04)	0.095	1.41 (1.07–1.85)	0.014	1.34 (1.02–1.77)	0.037
In-hospital outcome in Lanzhou cohort								
Any MACCE	60/1,182 (5.1%)	271/5,710 (4.7%)	1.07 (0.80–1.42)	0.629	0.92 (0.67–1.24)	0.593	0.93 (0.68–1.26)	0.652
New-onset HF	46/1,182 (3.9%)	202/5,710 (3.5%)	1.10 (0.79–1.52)	0.552	1.00 (0.70–1.42)	0.984	1.01 (0.70–1.44)	0.945
Non-fatal stroke	11/1,182 (0.9%)	52/5,710 (0.9%)	1.02 (0.50–1.89)	0.948	0.80 (0.39–1.51)	0.515	0.85 (0.41–1.60)	0.629
Non-fatal reinfarction	1/1,182 (0.1%)	8/5,710 (0.1%)	0.60 (0.03–3.29)	0.634	0.51 (0.03–3.02)	0.537	0.47 (0.02–2.93)	0.502
Non-fatal TVR	2/1,182 (0.2%)	17/5,710 (0.3%)	0.57 (0.09–1.98)	0.449	0.41 (0.06–1.47)	0.239	0.45 (0.07–1.61)	0.29
Length of stay (days)	7.00 (5.00–9.00)	7.00 (5.00–9.00)	−0.07 (−0.30–0.17)	0.577	−0.16 (−0.40–0.08)	0.19	−0.11 (−0.34–0.12)	0.334
Other adverse outcome in MIMIC-IV cohort								
7-day Delirium	173/608 (28.5%)	902/3,872 (23.3%)	1.35 (1.15–1.59)	<0.001	1.53 (1.29–1.81)	<0.001	1.40 (1.17–1.67)	<0.001
7-day AKI	403/608 (66.3%)	2,694/3,872 (69.6%)	1.01 (0.91–1.12)	0.878	1.04 (0.94–1.16)	0.439	1.05 (0.94–1.17)	0.365

1. Lanzhou cohort: Model 1: unadjusted; Model 2: adjusted for demographics (age, sex, Han ethnicity, medical insurance, urban residence), comorbidities and clinical presentation (CVD, COPD, SBP, heart rate, creatinine, infarct location, CS, Killip class IV); Model 3: additionally adjusted for treatment variables (aspirin, P2Y12i, timely reperfusion, IABP, ECMO).

2. MIMIC-IV cohort: Model 1: unadjusted; Model 2: adjusted for demographics (age, sex, race, medical insurance), comorbidities and clinical presentation (CCI, SBP, heart rate, respiratory rate); Model 3: additionally adjusted for treatment variables (aspirin, P2Y12i, mechanical ventilation, IABP, ECMO, PCI).

CCI, Charlson comorbidity index; CI, confidence interval; COPD, chronic obstructive pulmonary disease; CRRT, continuous renal replacement therapy; CS, cardiogenic shock; CVD, cardiovascular disease; ECMO, extracorporeal membrane oxygenation; IABP, intra-aortic balloon pump; PCI, percutaneous coronary intervention; P2Y12i, P2Y12 inhibitor; SBP, systolic blood pressure; SMuRF, standard modifiable cardiovascular risk factor.

a In-hospital binary clinical outcomes were analyzed using logistic regression models; continuous outcomes were analyzed using linear regression models.

**Figure 2:**
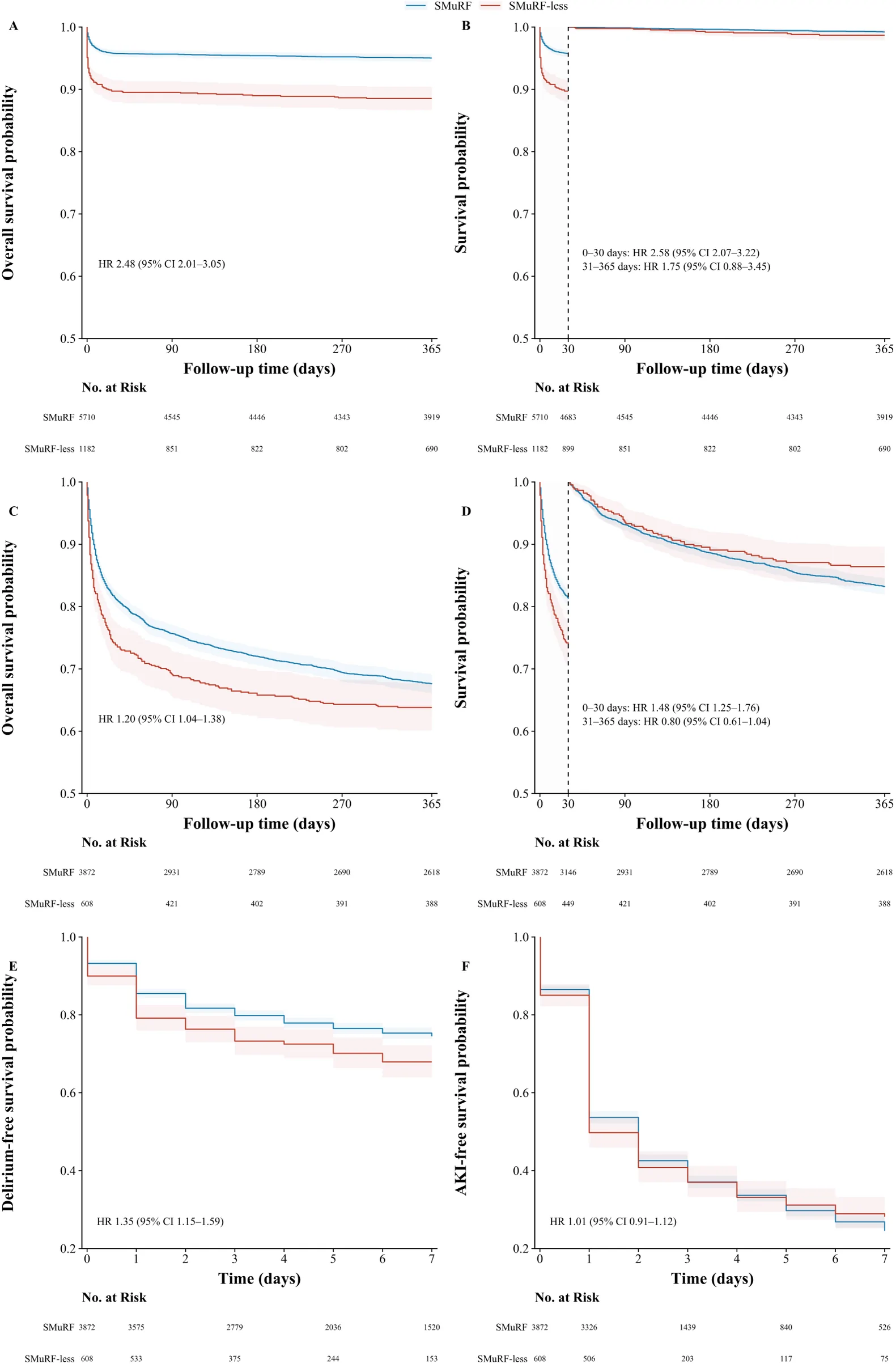
Kaplan–Meier survival curves for mortality and adverse outcomes in the Lanzhou and MIMIC-IV cohorts. **(A)** Kaplan–Meier curve for 1-year all-cause mortality in the Lanzhou cohort, **(B)** Landmark analysis curve for 1-year all-cause mortality in the Lanzhou cohort, **(C)** Kaplan–Meier curve for 1-year all-cause mortality in the MIMIC-IV cohort, **(D)** Landmark analysis curve for 1-year all-cause mortality in the MIMIC cohort, **(E)** Kaplan–Meier curve for 7-days delirium within in the MIMIC-IV cohort, and **(F)** Kaplan–Meier curve for 7-days acute kidney injury in the MIMIC-IV cohort. SMuRF-less status indicates the absence of all four standard modifiable cardiovascular risk factors.

### 3.4 MACCEs and other adverse outcomes

In the Lanzhou cohort, SMuRF-less status was not significantly associated with MACCEs (adjusted odds ratio [aOR], 0.93; 95% CI, 0.68–1.26), new-onset heart failure (aOR, 1.01; 95% CI, 0.70–1.44), non-fatal reinfarction (aHR, 0.47; 95% CI, 0.02–2.93), non-fatal stroke (aOR, 0.85; 95% CI, 0.41–1.60), or TVR (aOR, 0.45; 95% CI, 0.07–1.61) (all *P* > 0.05). No significant difference was observed in length of hospital stay (mean difference: −0.11 days, 95% CI, −0.34 to 0.12; *P* = 0.334) (Table 2). In the MIMIC-IV cohort, SMuRF-less patients had a higher risk of 7-day delirium (28.5% vs. 23.3%; aHR, 1.40; 95% CI, 1.17–1.67; *P* < 0.001). However, the risk of 7-day AKI was similar between groups (66.3% vs. 69.6%; aHR, 1.05; 95% CI, 0.94–1.17; *P* = 0.365).

### 3.5 Subgroup and sensitivity analyses

Figure 3 illustrates the subgroup analyses of the association between SMuRF-less status and 30-day mortality. Significant interactions were observed for Killip class, IABP use and ECMO use in the Lanzhou cohort (Figure 3A) (all *P* for interaction <0.05). The association was stronger among patients without Killip class IV and in the subgroups without IABP or ECMO use. No significant interaction by sex was observed in either cohort (both *P* for interaction >0.05). Among SMuRF-less patients, women had a higher 30-day mortality risk than men in the MIMIC-IV cohort (age-adjusted HR, 1.64; 95% CI, 1.19–2.24; *P* = 0.002), but not in the Lanzhou cohort (age-adjusted HR, 1.02; 95% CI, 0.68–1.53; *P* = 0.938) (Supplementary Table S5).

**Figure 3:**
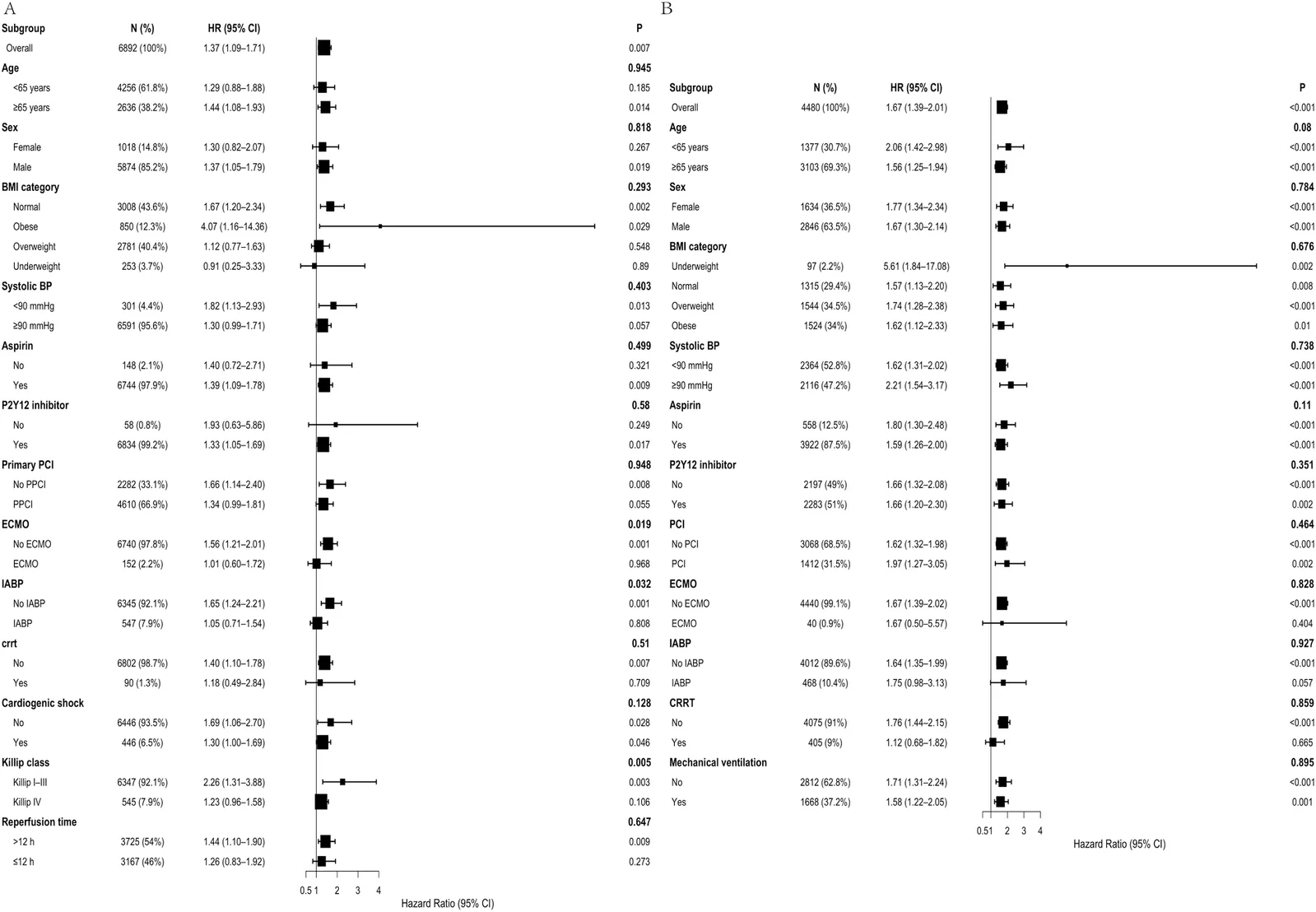
Subgroup analyses of the association between SMuRF-less status and 30-day all-cause mortality. **(A)** The Lanzhou cohort. Models were adjusted for age, sex, ethnicity, type of medical insurance, urban residence, history of cardiovascular disease, COPD, systolic blood pressure, heart rate, creatinine, infarct location, cardiogenic shock, Killip class IV, aspirin use, P2Y12 inhibitor use, reperfusion within 12 h, IABP, and ECMO, except the stratification variable. **(B)** The MIMIC-IV cohort. Models were adjusted for age, sex, race, insurance status, CCI, systolic blood pressure, heart rate, respiratory rate, creatinine, aspirin use, P2Y12 inhibitor use, PCI, early mechanical ventilation, IABP, and ECMO, except the stratification variable. CCI, Charlson comorbidity index; CI, confidence interval; COPD, chronic obstructive pulmonary disease; CRRT, continuous renal replacement therapy; ECMO, extracorporeal membrane oxygenation; HR, heart rate; IABP, intra-aortic balloon pump; P2Y12, platelet P2Y12 receptor; PCI, percutaneous coronary intervention; SMuRF, standard modifiable cardiovascular risk factor.

Sensitivity analyses using an expanded SMuRF definition that incorporated obesity yielded findings consistent with the primary analyses for 30-day mortality and other adverse outcomes (Supplementary Table S2). In the MIMIC-IV cohort, competing-risk analyses using Fine–Gray models, with death treated as a competing event, yielded results consistent with the primary analyses. SMuRF-less status remained associated with a higher risk of 7-day delirium (subdistribution hazard ratio [sHR], 1.36; 95% CI, 1.15–1.61; *P* < 0.001), but not with 7-day AKI (sHR, 1.03; 95% CI, 0.93–1.13; *P* = 0.572) (Supplementary Table S4). Further analyses of MAKE30 components in the MIMIC-IV cohort showed that SMuRF-less status was significantly associated with mortality but not with new renal replacement therapy or persistent renal dysfunction. In multinomial logistic regression analysis, SMuRF-less status was associated with a higher risk of death (aOR, 2.09; 95% CI, 1.62–2.71; *P* < 0.001), but not with new renal replacement therapy (aOR, 0.78; 95% CI, 0.35–1.73; *P* = 0.536) or persistent renal dysfunction (aOR, 0.88; 95% CI, 0.57–1.37; *P* = 0.575) (Supplementary Table S6).

### 3.6 Sequential attenuation analysis of clinical outcomes

Sequential attenuation analyses were performed in the MIMIC-IV cohort. For 30-day mortality, SMuRF-less status was associated with a higher risk after adjustment for demographic characteristics and comorbidities (aHR, 2.10; 95% CI, 1.76–2.51) (Figure 4A). Additional adjustment for metabolic, inflammatory, and hematologic markers slightly strengthened the association (aHR, 2.31; 95% CI, 1.92–2.78), corresponding to a 13.1% amplification. Further adjustment for hypoperfusion and organ or myocardial injury markers attenuated the association by 1.0% (aHR, 2.08; 95% CI, 1.73–2.51). The greatest attenuation was observed after additional adjustment for in-hospital treatments and organ-support interventions, resulting in a 41.8% reduction in the β coefficient (aHR, 1.54; 95% CI, 1.27–1.87).

**Figure 4:**
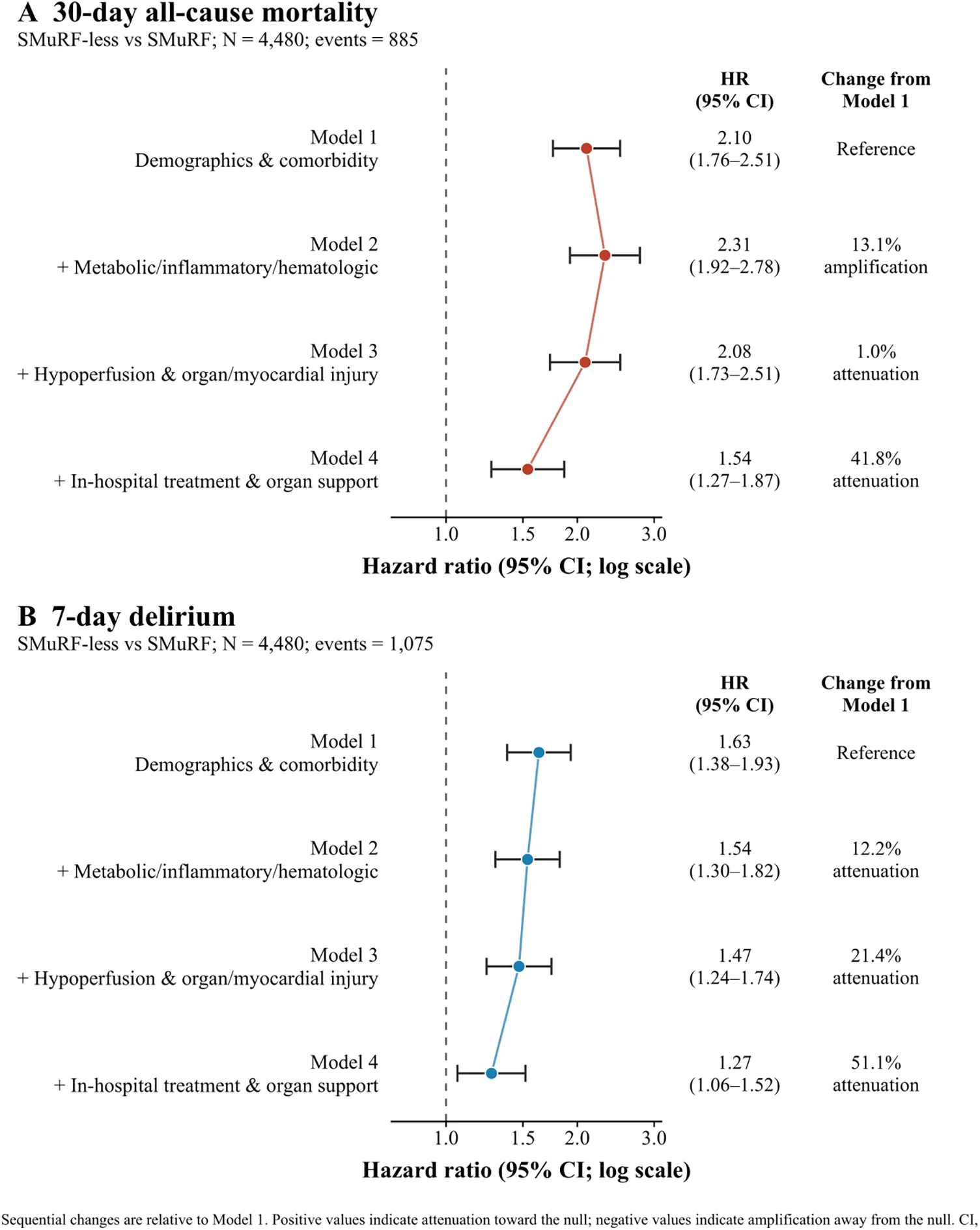
Sequential attenuation analysis of clinical outcomes in the MIMIC-IV cohort. **(A)** Associations of SMuRF-less status with 30-day all-cause mortality. **(B)** Associations of SMuRF-less status with 7-day delirium. Model 1 was adjusted for age, sex, race, insurance status, CCI, and COPD. Model 2 additionally included anion gap, WBC, activated partial thromboplastin time, platelet count, bicarbonate, potassium, fasting glucose, and hemoglobin. Model 3 further included systolic blood pressure, mean arterial pressure, heart rate, respiratory rate, lactate, creatinine, blood urea nitrogen, and CK-MB. Model 4 additionally included aspirin use, P2Y12 inhibitor use, PCI, ACEI, ARB, β-blocker, and statin use, as well as early mechanical ventilation, IABP, ECMO, and CRRT. Percentage changes in the β coefficient were calculated relative to Model 1. Positive values indicate attenuation, whereas negative values indicate amplification. ACEI, angiotensin-converting enzyme inhibitor; ARB, angiotensin receptor blocker; CCI, Charlson comorbidity index; CI, confidence interval; CK-MB, creatine kinase–MB; COPD, chronic obstructive pulmonary disease; CRRT, continuous renal replacement therapy; ECMO, extracorporeal membrane oxygenation; IABP, intra-aortic balloon pump; PCI, percutaneous coronary intervention; P2Y12, platelet P2Y12 receptor; WBC, white blood cell count; SMuRF, standard modifiable cardiovascular risk factor.

A similar pattern was observed for 7-day delirium (Figure 4B). The association between SMuRF-less status and delirium was significant after adjustment for demographic characteristics and comorbidities (aHR, 1.63; 95% CI, 1.38–1.93). Additional adjustment for metabolic, inflammatory, and hematologic markers attenuated the association by 12.2% relative to Model 1 (aHR, 1.54; 95% CI, 1.30–1.82). Further inclusion of hypoperfusion and organ or myocardial injury markers resulted in a cumulative attenuation of 21.4% (aHR, 1.47; 95% CI, 1.24–1.74). After additional adjustment for in-hospital treatments and organ-support interventions, the β coefficient was attenuated by 51.1%, although the association remained significant (aHR, 1.27; 95% CI, 1.06–1.52).

## 4 Discussion

Using data from two cohorts, this study yielded several important findings. First, compared with patients with at least one SMuRF, SMuRF-less patients had a significantly higher risk of 30-day mortality in both cohorts. Landmark analyses showed that the association was stronger during the first 30 days after admission. During days 31–365, the association was not significant in the Lanzhou cohort but remained significant after multivariable adjustment in the MIMIC-IV cohort. Second, beyond early mortality, SMuRF-less status was associated with a higher risk of 7-day delirium. Third, subgroup analyses suggested that the association between SMuRF-less status and 30-day mortality was stronger among patients not receiving mechanical circulatory support and among those with Killip class I–III. Finally, sequential attenuation analyses showed that additional adjustment for in-hospital treatments and organ-support interventions resulted in the greatest attenuation of the associations with 30-day mortality and 7-day delirium.

Several features of the STEMI patients and their clinical management are also noteworthy in both cohorts. Among patients with at least one SMuRF, the Lanzhou cohort was characterized predominantly by smoking, whereas the MIMIC-IV cohort showed a more metabolic risk profile. This contrast may reflect different epidemiological characteristics in the two cohorts. The prevalence of SMuRF-less STEMI was 17.2% in the Lanzhou cohort and 13.6% in the MIMIC-IV cohort, both broadly within the range reported in previous studies (20, 21). Additionally, the Lanzhou cohort showed a higher proportion of women in the SMuRF-less group (24.0% vs. 12.9%), consistent with prior reports of relative female over-representation in SMuRF-less STEMI populations (22, 23). This pattern may be partly attributable to the marked sex difference in smoking prevalence, which was substantially lower in women than in men (4.5% vs. 64.7%). However, in the MIMIC-IV cohort, the sex distribution was similar between the SMuRF-less and SMuRF groups (35.4% vs. 36.6%), as were smoking rates between women and men (10.5% vs. 12.0%). Consistent with previous findings, we observed a higher risk of 30-day mortality in women than in men in the MIMIC-IV cohort, even after adjustment for age (5). SMuRF-less patients in the MIMIC-IV cohort were 5.8 years younger, differing from some previous reports, while their lower comorbidity burden was consistent with previous studies (5, 23–25). This may represent a subgroup in whom disease onset is more closely related to unmeasured or inherited susceptibility than cumulative risk exposures (26). Consistent with previous reports, SMuRF-less patients in our analyses were less likely to receive GDMT and timely reperfusion, with longer symptom-to-device times observed in the Lanzhou cohort (5, 24).

Our findings show that SMuRF-less patients with STEMI have a poorer prognosis, characterized by an increased risk of early mortality, consistent with evidence from large-scale clinical registries (23, 27). Notably, in the MIMIC-IV cohort, SMuRF-less status was not significantly associated with mortality during days 31–365 in the unadjusted analysis, but a significant association emerged after multivariable adjustment. However, a recent study suggests that patients with multiple SMuRFs may face a higher long-term risk of recurrent ischemic events after hospital discharge (28). In the Lanzhou cohort, SMuRF-less status was not significantly associated with in-hospital MACCEs. These findings raise the possibility that SMuRF-less status may be associated with a lower risk of certain recurrent ischemic events, but not necessarily with a lower risk of mortality (4).

By evaluating two cohorts from different regions, this study provides further evidence for the SMuRF-less paradox (29). Despite the absence of conventional risk factors, SMuRF-less patients presented with greater hemodynamic instability. Compared with patients with SMuRFs, they had lower SBP, higher heart rates, and approximately twice the rate of cardiogenic shock. Notably, the significantly higher risk of 7-day delirium observed in the MIMIC-IV cohort provides further evidence of the neurological vulnerability associated with this phenotype. These findings were accompanied by increased use of mechanical ventilation and vasopressor support (29), further reflecting the greater clinical severity of SMuRF-less patients.

In this study, SMuRF-less patients in the Lanzhou cohort had a higher prevalence of anterior myocardial infarction, consistent with findings from Swedish and Australian registries (4, 5). Similarly, significantly elevated CK-MB levels in the MIMIC-IV cohort suggested more extensive myocardial injury. Evidence from the DANAMI-3 sub-study further suggests that SMuRF-less patients have larger infarct sizes and reduced myocardial salvage compared to those with traditional risk factors (30). Collectively, these findings suggest that SMuRF-less status may exhibit a more severe myocardial infarction phenotype characterized by greater myocardial injury (5, 30), potentially related to a lack of ischemic preconditioning or unrecognized genetic predispositions (31). Differences in clinical management may also contribute to the observed adverse outcomes among SMuRF-less patients. In our cohorts, despite greater clinical severity, SMuRF-less patients were less likely to receive some guideline-directed medical therapies and reperfusion therapies. These findings align with large-scale registry data (24). These treatment differences may reflect differences in clinical risk assessment, treatment indications, or contraindications, which could not be fully evaluated in the present study (32, 33).

In the MIMIC-IV cohort, sequential attenuation analyses were performed to explore factors potentially contributing to the increased risks of 30-day mortality and 7-day delirium in SMuRF-less patients. For 30-day mortality, additional adjustment for hypoperfusion and organ or myocardial injury markers resulted in relatively little attenuation of the association. By contrast, the inclusion of in-hospital treatment and organ-support variables substantially attenuated the association, reducing the β coefficient by 41.8% relative to Model 1. This pattern suggests that treatment-related factors may partly account for the observed association with early mortality. However, the observed attenuation does not establish a causal mediating role of these interventions, as treatment decisions and organ-support requirements are closely related to disease severity and clinical indications (9, 34). The stronger association observed among patients not receiving IABP or ECMO support further highlights the potential relevance of treatment-related factors to early mortality risk. For 7-day delirium, the observed attenuation suggests a possible contribution of metabolic disturbance, systemic inflammation, and acute physiological stress to neurological vulnerability (35, 36). The greatest attenuation occurred after additional adjustment for treatment and organ-support variables, reducing the β coefficient by 51.1% relative to Model 1. However, the association remained significant, suggesting that these measured factors did not fully account for the observed association. Collectively, these findings suggest that the adverse prognosis of SMuRF-less patients may reflect a combination of clinical severity, treatment-related factors, and underlying biological susceptibility (24, 37–39).

## 5 Strengths and limitations

### 5.1 Strengths

The major strength of this study is the inclusion of two large, independent, and well-phenotyped STEMI cohorts: the Lanzhou cohort from China and the MIMIC-IV cohort from the United States. Unlike most previous studies that focused primarily on mortality, we expanded the outcome spectrum to include acute complications such as delirium and adverse renal events, thereby providing more comprehensive evidence to inform early risk assessment and clinical management in SMuRF-less patients. Furthermore, we conducted sensitivity analyses incorporating obesity as the fifth risk factor, addressing a potential limitation of the traditional four-risk-factor framework. The inclusion of ethnic minority patients (7.9%) in the Lanzhou cohort also contributed to the diversity of the study population. Finally, leveraging the detailed clinical data available in MIMIC-IV, we performed sequential attenuation analyses to explore how adjustment for biological and treatment-related factors influenced the observed associations with clinical outcomes, providing potential mechanistic insights.

### 5.2 Limitations

Several limitations should be acknowledged. First, despite extensive adjustment for a wide range of covariates, the observational design cannot completely exclude the impact of potential confounders, such as unmeasured exposures or differences in specific biomarkers. Second, the definition of SMuRF status relied heavily on extraction from electronic medical records. In retrospective databases, particularly MIMIC-IV, underreporting of smoking history or misclassification of hyperlipidemia may occur, especially in acute patients without routine lipid testing, which could lead to an erroneous categorization of some patients into the SMuRF-less group. Third, this study lacked data on certain emerging inflammatory markers (e.g., high-sensitivity C-reactive protein), polygenic risk scores, and detailed socioeconomic status. These factors may contribute to the prognosis among SMuRF-less patients, and their absence limits a comprehensive elucidation of deeper biological mechanisms.

## 6 Conclusion

In this study, SMuRF-less status was consistently associated not only with increased early mortality in both cohorts but also with a higher risk of 7-day delirium in the MIMIC-IV cohort. Treatment-related factors may partly contribute to the observed associations, highlighting the importance of early clinical risk assessment and management. Further studies are needed to identify the underlying biological mechanisms.

## Data Availability

The original contributions presented in the study are included in the article/Supplementary Material, further inquiries can be directed to the corresponding authors.
