# Supplementary material for: The SMuRF-less paradox in ST-segment-elevation myocardial infarction: a retrospective hospital-based two-cohort study
Source: Front Cardiovasc Med. 2026 Oct 30;13:1955087. doi: 10.3389/fcvm.2026.1955087 (PMC13759292; doi:10.3389/fcvm.2026.1955087)
Supplement: Supplementary file 1 [file Datasheet1.docx]

**Supplementary Material**

## Table S1. Related delay in patients under PPCI within onset-of-symptoms to admission ≤ 24 h (n = 4 310)

|  | Total | SMuRF-less | SMuRF | P value |
| --- | --- | --- | --- | --- |
|  | N = 4310 | N = 745 | N = 3565 |  |
| Total ischaemic time delays | | | | |
| Symptoms-to-thrombolysis | | | | |
| < 3 h | 556 (12.9%) | 90 (12.1%) | 466 (13.1%) | 0.46 |
| Median min | 183 (105-288) | 195 (107-313) | 180 (104-285) | 0.17 |
| Missing % | 190 ( 4.4%) | 25 ( 3.4%) | 165 ( 4.6%) | 0.12 |
| Symptoms-to-device | | | | |
| < 2 h | 145 ( 3.4%) | 22 ( 3.0%) | 123 ( 3.5%) | 0.49 |
| Median min | 429 (274-664) | 456 (294-712) | 425 (271-655) | 0.006 |
| Missing % | 486 (11%) | 89 (12%) | 397 (11%) | 0.52 |
| Symptoms-to-start of reperfusion therapy : thrombolysis or PPCI | | | | |
| < 3 h | 951 (22.1%) | 150 (20.1%) | 801 (22.5%) | 0.16 |
| < 12 h | 3 130 (72.6%) | 536 (71.9%) | 2 594 (72.8%) | 0.65 |
| Median min | 313 (178-577) | 335 (185-602) | 310 (175-570) | 0.11 |
| Missing % | 539 (12.5%) | 87 (11.7%) | 452 (12.7%) | 0.45 |
| Patient-related delays | | | | |
| Symptoms-to-FMC |  |  |  |  |
| < 2 h | 1 493 (34.6%) | 257 (34.5%) | 1 236 (34.7%) | 0.93 |
| Median min | 175 (80-390) | 180 (80-410) | 173 (80-386) | 0.68 |
| Missing % | 203 ( 4.7%) | 22 ( 3.0%) | 181 ( 5.1%) | 0.013 |
| Delays in the medical system | | | | |
| FMC-to-device |  |  |  |  |
| < 90 min | 959 (22.3%) | 153 (20.5%) | 806 (22.6%) | 0.22 |
| < 120 min | 1 375 (31.9%) | 219 (29.4%) | 1 156 (32.4%) | 0.11 |
| Median min | 184 (90-302) | 192 (95-311) | 183 (89-300) | 0.092 |
| Missing % | 464 (10.8%) | 84 (11.3%) | 380 (10.7%) | 0.62 |
| Bypassing the CCU | 3 761 (87.3%) | 678 (91.0%) | 3 083 (86.5%) | <0.001 |
| Bypassing the ED | 2 009 (46.6%) | 365 (49.0%) | 1 644 (46.1%) | 0.15 |
| Dual bypass | 1 789 (41.5%) | 337 (45.2%) | 1 452 (40.7%) | 0.023 |
| Door of PCI hospital-to-device |  |  |  |  |
| < 90 min | 2 815 (65.3%) | 488 (65.5%) | 2 327 (65.3%) | 0.9 |
| Median min | 60 (33-95) | 58 (33-93) | 60 (33-95) | 0.63 |
| Missing % | 406 ( 9.4%) | 78 (10.5%) | 328 ( 9.2%) | 0.28 |
| Abbreviations: PPCI, primary percutaneous coronary intervention; SMuRF, standard modifiable cardiovascular risk factors; FMC, first medical contact; CCU, coronary care unit; ED, emergency department. | | | | |

## Table S2. Sensitivity analysis of SMuRF-less status and clinical outcomes incorporating obesity (BMI ≥ 30 kg/m²) as an additional risk factor

| Outcomes | SMuRF-less（n,%) | SMuRF （n,%) | Hazard ratio (95% CI)^*^ | | | | | |
| --- | --- | --- | --- | --- | --- | --- | --- | --- |
|  |  |  | Model 1 | P value | Model 2 | P value | Model 3 | P value |
| 30-day Mortality | | | | | | | | |
| Lanzhou cohort | 118/1147 (10.3%) | 234/5745 (4.1%) | 2.64 (2.12–3.30) | <0.001 | 1.47 (1.17–1.84) | <0.001 | 1.40 (1.12–1.76) | 0.003 |
| MIMIC-IV cohort | 118/439 (26.9%) | 767/4041 (19.0%) | 1.51 (1.24–1.83) | <0.001 | 1.78 (1.45–2.17) | <0.001 | 1.63 (1.33–1.99) | <0.001 |
| 31-365-day Mortality |  |  |  |  |  |  |  |  |
| Lanzhou cohort | 9/869 (1.0%) | 36/4713 (0.8%) | 1.40 (0.67–2.91) | 0.366 | 1.26 (0.60–2.66) | 0.537 | 1.31 (0.62–2.75) | 0.483 |
| MIMIC-IV cohort | 52/321 (16.2%) | 539/3274 (16.5%) | 0.99 (0.74–1.31) | 0.931 | 1.54 (1.15–2.06) | 0.004 | 1.46 (1.09–1.96) | 0.012 |
| In-hospital outcome in Lanzhou cohort | | | | | | | | |
| Any MACCE | 59/1147 (5.1%) | 272/5745 (4.7%) | 1.09 (0.81–1.45) | 0.554 | 0.93 (0.67–1.25) | 0.625 | 0.94 (0.68–1.27) | 0.693 |
| New-onset HF | 45/1147 (3.9%) | 203/5745 (3.5%) | 1.11 (0.79–1.53) | 0.518 | 1.00 (0.69–1.42) | 0.999 | 1.02 (0.70–1.45) | 0.935 |
| Non-fatal stroke | 11/1147 (1.0%) | 52/5745 (0.9%) | 1.06 (0.52–1.96) | 0.861 | 0.83 (0.40–1.56) | 0.581 | 0.87 (0.42–1.65) | 0.693 |
| Non-fatal reinfarction | 1/1147 (0.1%) | 8/5745 (0.1%) | 0.63 (0.03–3.41) | 0.659 | 0.53 (0.03–3.14) | 0.562 | 0.48 (0.02–3.00) | 0.516 |
| Non-fatal TVR | 2/1147 (0.2%) | 17/5745 (0.3%) | 0.59 (0.09–2.06) | 0.479 | 0.42 (0.07–1.53) | 0.258 | 0.46 (0.07–1.67) | 0.312 |
| Length of stay (days) | 7.00 (5.00–9.00) | 7.00 (5.00–9.00) | -0.11 (-0.35–0.13) | 0.384 | -0.20 (-0.45–0.04) | 0.102 | -0.15  (-0.39–0.08) | 0.194 |
| Other adverse outcome in MIMIC-IV cohort | | | | | | | | |
| 7-day Delirium | 120/439 (27.3%) | 955/4041 (23.6%) | 1.26 (1.04–1.52) | 0.018 | 1.40 (1.15–1.70) | <0.001 | 1.33 (1.09–1.62) | 0.005 |
| 7-day AKI | 279/439 (63.6%) | 2818/4041 (69.7%) | 0.91 (0.81–1.03) | 0.150 | 0.93 (0.82–1.05) | 0.257 | 0.94 (0.83–1.07) | 0.355 |
| 1.Lanzhou cohort: Model 1: unadjusted; Model 2: adjusted for demographics (age, sex, Han ethnicity, medical insurance, urban residence), comorbidities and clinical presentation (CVD, COPD, SBP, heart rate, creatinine, infarct location, CS, Killip class); Model 3: additionally adjusted for treatment variables (aspirin, P2Y12i, timely reperfusion, IABP, ECMO) ; 2.MIMIC-IV cohort: Model 1: unadjusted; Model 2: adjusted for demographics (age, sex, race, medical insurance), comorbidities and clinical presentation (CCI, SBP, heart rate, respiratory rate, creatinine); Model 3: additionally adjusted for treatment variables (aspirin, P2Y12i, mechanical ventilation, IABP, ECMO, PCI).  * In-hospital binary clinical outcomes including MACCEs and MAKE30 were analyzed using logistic regression models; continuous outcomes were analyzed using linear regression models. Abbreviations: BMI, body mass index; CCI, Charlson Comorbidity Index; CI, confidence interval; COPD, chronic obstructive pulmonary disease; CS, cardiogenic shock; CVD, cardiovascular disease; ECMO, extracorporeal membrane oxygenation; IABP, intra-aortic balloon pump; PCI, percutaneous coronary intervention; P2Y12i, P2Y12 inhibitor; SBP, systolic blood pressure; SMuRF, standard modifiable cardiovascular risk factors. | | | | | | | | |

## Table S3. Sensitivity analysis using a restrictive STEMI definition in the MIMIC-IV cohort

| Outcomes | Hazard ratio (95% CI) | | | | | |
| --- | --- | --- | --- | --- | --- | --- |
|  | Model 1 | P value | Model 2 | P value | Model 3 | P value |
| Traditional SMuRF definition | | | | | | |
| 30-day Mortality | 1.23 (1.00–1.51) | 0.046 | 1.47 (1.19–1.82) | <0.001 | 1.39 (1.11–1.73) | 0.003 |
| 31-365-day Mortality | 0.61 (0.43–0.86) | 0.004 | 1.00 (0.70–1.43) | 0.994 | 0.93 (0.65–1.33) | 0.687 |
| 7-day Delirium | 1.26 (1.05–1.50) | 0.011 | 1.39 (1.16–1.68) | <0.001 | 1.24 (1.02–1.50) | 0.028 |
| 7-day AKI | 1.05 (0.93–1.19) | 0.406 | 1.04 (0.91–1.18) | 0.586 | 1.04 (0.92–1.19) | 0.535 |
| SMuRF definition incorporating obesity (BMI ≥ 30 kg/m²) | | | | | | |
| 30-day Mortality | 1.35 (1.07–1.70) | 0.011 | 1.53 (1.21–1.94) | <0.001 | 1.43 (1.12–1.82) | 0.004 |
| 31-365-day Mortality | 0.73 (0.50–1.08) | 0.112 | 1.02 (0.68–1.51) | 0.937 | 0.93 (0.62–1.39) | 0.717 |
| 7-day Delirium | 1.23 (1.00–1.51) | 0.055 | 1.33 (1.07–1.64) | 0.010 | 1.21 (0.97–1.50) | 0.086 |
| 7-day AKI | 0.98 (0.85–1.13) | 0.775 | 0.94 (0.81–1.09) | 0.448 | 0.95 (0.82–1.10) | 0.486 |
| Model 1: unadjusted; Model 2: adjusted for demographics (age, sex, race, medical insurance), comorbidities and clinical presentation (COPD, CCI, SBP, HR, respiratory rate); Model 3: additionally adjusted for treatment variables (aspirin, P2Y12i, mechanical ventilation, IABP, ECMO, PCI).  Abbreviations: BMI, body mass index; CCI, Charlson Comorbidity Index; CI, confidence interval; COPD, chronic obstructive pulmonary disease; ECMO, extracorporeal membrane oxygenation; HR, heart rate; IABP, intra-aortic balloon pump; PCI, percutaneous coronary intervention; P2Y12i, P2Y12 inhibitor; SBP, systolic blood pressure; SMuRF, standard modifiable cardiovascular risk factor. | | | | | | |

Table S4. Fine–Gray Competing-Risk Analyses of 7-Day Delirium and AKI in the MIMIC-IV cohort

| Outcomes | Subdistribution hazard ratios (sHRs, 95% CI) | | | | | |
| --- | --- | --- | --- | --- | --- | --- |
|  | Model 1 | P value | Model 2 | P value | Model 3 | P value |
| Traditional SMuRF definition | | | | | | |
| 7-day delirium | 1.30 (1.11–1.52) | 0.001 | 1.47 (1.24–1.73) | <0.001 | 1.36 (1.15–1.61) | <0.001 |
| 7-day AKI | 0.98 (0.89–1.08) | 0.663 | 1.01 (0.92–1.11) | 0.843 | 1.03 (0.93–1.13) | 0.572 |
| SMuRF definition incorporating obesity (BMI ≥ 30 kg/m²) | | | | | | |
| 7-day delirium | 1.22 (1.01–1.47) | 0.036 | 1.35 (1.12–1.63) | 0.002 | 1.29 (1.07–1.55) | 0.007 |
| 7-day AKI | 0.90 (0.81–1.01) | 0.072 | 0.92 (0.82–1.03) | 0.150 | 0.94 (0.84–1.05) | 0.270 |
| Model 1: unadjusted; Model 2: adjusted for demographics (age, sex, race, medical insurance), comorbidities and clinical presentation (COPD, CCI, SBP, heart rate, respiratory rate); Model 3: additionally adjusted for treatment variables (aspirin, P2Y12i, mechanical ventilation, IABP, ECMO, PCI). Abbreviations: AKI, acute kidney injury; BMI, body mass index; CCI, Charlson Comorbidity Index; CI, confidence interval; COPD, chronic obstructive pulmonary disease; ECMO, extracorporeal membrane oxygenation; IABP, intra-aortic balloon pump; PCI, percutaneous coronary intervention; P2Y12i, P2Y12 inhibitor; SBP, systolic blood pressure; RRT, renal replacement therapy; SMuRF, standard modifiable cardiovascular risk factor. | | | | | | |

Table S5. Sex-Specific 30-Day Mortality Among SMuRF-Less Patients in the Lanzhou and MIMIC-IV Cohorts

| Cohort | 30-day mortality | | | Hazard ratio (95% CI) | | | |
| --- | --- | --- | --- | --- | --- | --- | --- |
|  | Crude, n/N (%) | KM estimate (95% CI) | Log-rank test | Crude model | P value | Age-adjusted model | P value |
| Lanzhou | | | | | | | |
| Male | 86/898 (9.58%) | 9.8% (7.8–11.7) | 0.295 | 1 (Reference) | 0.295 | 1 (Reference) | 0.938 |
| Female | 33/284 (11.62%) | 11.8% (7.9–15.6) |  | 1.24 (0.83–1.85) |  | 1.02 (0.68–1.53) |  |
| MIMIC-IV | | | | | | | |
| Male | 85/393 (21.63%) | 21.6% (17.5–25.6) | <0.001 | 1 (Reference) | <0.001 | 1 (Reference) | 0.002 |
| Female | 74/215 (34.42%) | 34.4% (27.8–40.5) |  | 1.76 (1.29–2.40) |  | 1.64 (1.19–2.24) |  |

Table S6. Multinomial Analysis of 30-day renal outcomes and death in the MIMIC-IV cohort

| Outcomes | Hazard ratio (95% CI) | | | | | |
| --- | --- | --- | --- | --- | --- | --- |
|  | Model 1 | P value | Model 2 | P value | Model 3 | P value |
| Traditional SMuRF definition | | | | | | |
| None | 1.00 | | 1.00 | | 1.00 | |
| Death | 1.47 (1.21–1.80) | <0.001 | 2.36 (1.85–3.01) | <0.001 | 2.09 (1.62–2.71) | <0.001 |
| New RRT | 0.33 (0.16–0.67) | 0.002 | 0.80 (0.37–1.77) | 0.590 | 0.78 (0.35–1.73) | 0.536 |
| Persistent renal dysfunction | 0.92 (0.61–1.39) | 0.682 | 0.89 (0.58–1.37) | 0.591 | 0.88 (0.57–1.37) | 0.575 |
| SMuRF definition incorporating obesity (BMI ≥ 30 kg/m²) | | | | | | |
| None | 1.00 | | 1.00 | | 1.00 | |
| Death | 1.49 (1.19–1.87) | <0.001 | 2.20 (1.68–2.88) | <0.001 | 1.99 (1.50–2.64) | <0.001 |
| New RRT | 0.11 (0.03–0.46) | 0.002 | 0.30 (0.07–1.24) | 0.096 | 0.29 (0.07–1.23) | 0.093 |
| Persistent renal dysfunction | 0.95 (0.59–1.52) | 0.818 | 0.93 (0.57–1.51) | 0.776 | 0.92 (0.56–1.50) | 0.742 |
| Model 1: unadjusted; Model 2: adjusted for demographics (age, sex, race, medical insurance), comorbidities and clinical presentation (CCI, SBP, heart rate, respiratory rate, creatinine); Model 3: additionally adjusted for treatment variables (aspirin, P2Y12i, mechanical ventilation, IABP, ECMO, PCI). Abbreviations: BMI, body mass index; CCI, Charlson Comorbidity Index; CI, confidence interval; ECMO, extracorporeal membrane oxygenation; IABP, intra-aortic balloon pump; PCI, percutaneous coronary intervention; P2Y12i, P2Y12 inhibitor; SBP, systolic blood pressure; SMuRF, standard modifiable cardiovascular risk factor. | | | | | | |
